# Functional Impairment of Microglia Coincides with Beta-Amyloid Deposition in Mice with Alzheimer-Like Pathology

**DOI:** 10.1371/journal.pone.0060921

**Published:** 2013-04-08

**Authors:** Grietje Krabbe, Annett Halle, Vitali Matyash, Jan L. Rinnenthal, Gina D. Eom, Ulrike Bernhardt, Kelly R. Miller, Stefan Prokop, Helmut Kettenmann, Frank L. Heppner

**Affiliations:** 1 Max Delbrueck Center for Molecular Medicine (MDC), Berlin, Germany; 2 Department of Neuropathology, Charité-Universitätsmedizin Berlin, Berlin, Germany; 3 Center of Advanced European Studies and Research (caesar), Bonn, Germany; Charité-Universitätsmedizin Berlin, Germany

## Abstract

Microglial cells closely interact with senile plaques in Alzheimer’s disease and acquire the morphological appearance of an activated phenotype. The significance of this microglial phenotype and the impact of microglia for disease progression have remained controversial. To uncover and characterize putative changes in the functionality of microglia during Alzheimer’s disease, we directly assessed microglial behavior in two mouse models of Alzheimer’s disease. Using *in vivo* two-photon microscopy and acute brain slice preparations, we found that important microglial functions - directed process motility and phagocytic activity - were strongly impaired in mice with Alzheimer’s disease-like pathology compared to age-matched non-transgenic animals. Notably, impairment of microglial function temporally and spatially correlated with Aβ plaque deposition, and phagocytic capacity of microglia could be restored by interventionally decreasing amyloid burden by Aβ vaccination. These data suggest that major microglial functions progressively decline in Alzheimer’s disease with the appearance of Aβ plaques, and that this functional impairment is reversible by lowering Aβ burden, e.g. by means of Aβ vaccination.

## Introduction

Microglial cells execute important functions in the brain. They constantly survey their surrounding and react to acute tissue injuries [1], [2]. In the healthy brain, microglia contact synapses for seconds, which can be prolonged up to one hour upon acute injury and eventually leads to the disappearance of the contacted neuronal structure [3], [4]. Microglia are also involved in shaping adult hippocampal neurogenesis through phagocytosis of apoptotic newborn neuroblasts [5]. Together, these data highlight that so-called “resting” microglia in the healthy brain are able to modify their environment, e.g. by their intrinsic phagocytic activity [6].

In Alzheimer’s disease (AD), microglia are attracted to β-amyloid (Aβ) plaques, produce elevated levels of proinflammatory cytokines and reactive oxygen species, and exhibit a change in morphology [7]–[11]. These phenotypical and morphological changes of microglia are commonly interpreted as an “activated” state [12], [13]. However, microglial activation is not an all-or-none process and the morphology of microglial cells does not necessarily match their functional state [14], [15].

A number of *in vitro* studies have not only shown that administration of Aβ stimulates the production of inflammatory cytokines and activation markers in microglial cells [8] but also triggers its uptake by receptor-mediated phagocytosis [16]. Further studies found that microglial cells internalize soluble, protofibrillar and fibrillar forms of Aβ [9], [17], [18] *in vitro* and *in vivo* by several mechanisms such as macropinocytosis or endocytosis, highlighting the general aptitude of microglia to remove Aβ. However, the question of whether resident microglia restrict Aβ plaque growth in AD has remained controversial, as the recruitment of microglia to Aβ plaques does not seem to result in their degradation [13], [18]. More importantly, we have recently found that temporary ablation of microglia has no effect on the formation and maintenance of Aβ plaques [19]. Interestingly, expression of receptors and enzymes involved in microglial Aβ-uptake and degradation is progressively downregulated in a transgenic mouse model of Alzheimer’s disease [20]. Thus, it has been suggested that microglia are possibly less efficient in removing and degrading Aβ at later stages of Alzheimer’s disease and become dysfunctional [13], [20].

In order to characterize putative progressive AD-associated changes in microglia not only at an expression level, but also functionally, we directly assessed and quantified microglial phagocytic and directed motile function in AD mouse models using two-photon microscopy and acute cerebral slice preparations. Furthermore, we reduced Aβ plaque burden in an Alzheimer’s disease mouse model by administering the Aβ-specific antibody Ab9 and assessed whether this interventional treatment restores AD-associated functional changes in microglia. Our data obtained from two different mouse models of Alzheimer’s disease reveals further insight into specific changes of microglial behavior during Alzheimer’s disease and suggest that Aβ plaque deposition and microglial function are closely interrelated.

## Materials and Methods

### Ethics Statement

All procedures involving handling of living animals were performed in accordance with the German animal protection law and were approved by the regional offices for health and social services in Berlin.

### Mouse Models

*APPPS1* mice, a transgenic mouse model of cerebral amyloidosis expressing human APP with the Swedish mutation (KM670/671NL) and human mutated PS1 (PS1-L166P) [21] were kindly provided by Mathias Jucker, University of Tübingen, Germany. *APP23* mice, which express APP with the Swedish mutation [22] were provided by Novartis, Basel, Switzerland. For two-photon imaging experiments *Cx3cr1* ^gfp/gfp^ mice [23] were crossbred to *APPPS1* mice and resulting offspring heterozygous for *Cx3cr1*GFP and/or for the *APPPS1* transgenes was used for imaging experiments. *Cx3cr1* ^gfp/gfp^ mice were kindly provided by Frank Kirchhoff, University of Saarland, Germany. All mice were kept under standard housing conditions with a 12 h light/dark cycle and access to standard food and water *ad libitum.* Throughout this study transgene-negative littermates were used as controls except when using *APP23* mice, where age-matched wildtype animals served as controls. Female and male mice were included in the experiments in an about equal distribution.

### Primary Microglial Cell Culture

Microglial cultures were prepared from cerebral cortex of 1–3 day old *APPPS1* and wildtype mice as previously described [24] and cultured in DMEM supplemented with 10% fetal calf serum, 2 mM L-glutamine, 100 units/ml penicillin and 100 µg/ml streptomycin. After establishment of an astrocytic monolayer, medium was additionally supplemented with 30% L929-conditioned DMEM (M-CSF-secreting mouse fibroblast cell line) to stimulate microglial proliferation. After separating microglia from the underlying astrocytic layer by shaking for one hour at 100 rpm, cells were seeded on glass coverslips at a density of 5×10^4^/cover slip. Cultures were used for experiments one day after plating. Cell-culture media and supplements were purchased from PAA Laboratories GmbH (Cölbe, Germany).

### Vaccination Paradigm

Ab9 (mouse anti Aβ aa. 1–16 IgG2a κ, QED Bioscience Inc.) or total nonspecific mouse IgG (control, SLM66; Equitech Bio) were injected intraperitoneally at 500 µg twice a week for 6 weeks in age-matched *APPPS1* mice (n = 3 per group) or WT (n = 2 per group) starting at 170 days of age.

### Preparation of Acute Brain Slices

Mice were decapitated and brains were carefully removed and washed in artificial cerebrospinal fluid (aCSF) containing (in mM): NaCl 134; KCl 2.5; MgCl_2_ 1.3; CaCl_2_ 2; K_2_HPO_4_ 1.25; NaHCO_3_ 26; D-glucose 10; pH 7.4. The buffer solution was saturated with carbogen (95% O_2_, 5% CO_2_). 130 µm thick coronal slices were prepared using a vibratome (Microm, Walldorf, Germany) at 4°C, and were kept in brain slice buffer at room temperature (21–25°C) for 2 h until the phagocytosis experiment was performed.

### Assessment of Phagocytosis

To quantify microglial phagocytic activity in acute brain slices, experiments were performed in *APPPS1* and non-transgenic littermates at 7–9 week and at 4 and 9 month (n = 3–4 per age group and genotype) of age, as well as in 20 month old *APP23* mice and aged-matched wildtype mice (n = 3 per genotype). Acute brain slices or primary microglial cell cultures (3 independent experiments) were incubated with a suspension of FCS-coated Yellowgreen fluorescent carboxylated microspheres (3 µm diameter, Polysciences Europe GmbH) at a concentration of 1.7×10^7^ microspheres/ml for 60 min (slices) or 30 min (cell culture) at 37°C, intensively washed and finally fixed with 4% paraformaldehyde. Brain sections and microglial cell cultures were stained with anti-Iba-1 and anti-Aβ (4G8) antibodies to visualize microglia and Aβ plaques, respectively.

### Immunohistochemistry

Fixed brain slices and primary microglial cells were incubated with 0.75 µg/ml anti-Iba-1 antibody (Wako) and 0.5 µg/ml 4G8 antibody (Signet). 6.25 µg/ml donkey anti-rabbit Cy3, 2.5 µg/ml donkey anti-mouse DyLight 649 (Jackson ImmunoResearch) or 4 µg/ml Alexa Fluor 568 goat anti rabbit (Invitrogen) secondary antibodies were used. After washing, slices were incubated for 30 min with Hoechst 33258 (Sigma-Aldrich; 1∶10000) or DRAQ5 (Cell Signalling Technology; 1∶1000) in 0.1 M PB and mounted in Aqua polymount for further analysis using confocal microscopy.

### Confocal Microscopy and Quantification of Phagocytosis

Confocal laser scanning microscopy was performed on Leica SPE and Zeiss LSM5 Exciter confocal microscopes with LAS AF and ZEN 2008 software, respectively. In brain sections derived from acute brain slices z-stacks of 20 µm thickness were performed using a 40x objective with a step size of 1 µm beginning from the top of the slice, where the microspheres are located. Beads per cell were counted using Image J MacBiophotonics cell counter plugin ensuring that only beads inside a cell were counted as positive. The phagocytic index was determined by assessing the percentage of cells which contained 0, 1–4, 5–7, 8–10 and >10 microspheres per cell. The percentage of cells in each group was multiplied by the corresponding grade of phagocytosis (1–4∶1, 5–7∶2, 8–10∶3, >10∶4). The sum of the products in each group was then termed and displayed as phagocytic index [25]. 4–15 ROIs (i.e. fields of view) were analyzed per animal.

### Plaque Load Assessment

Brain sections were incubated in 0.001% Thiazine Red (Sigma) solution in 0.1 M PB, thereby labeling the β-sheet structure of dense core plaques [26]. Thiazine Red-positive plaques were quantified by scanning cerebral sections with a Nikon Ti Epsilon microscope using a TRITC FL-filter set and fixed acquisition settings. Large images of whole coronal brain section were obtained by stitching single images using NIS Elements software. Coverage of respective brain area (cortex, hippocampus, cerebellum) by Thiazine Red-positive plaques was quantified by analyzing images using Image J with a fixed intensity threshold. Staining artifacts were manually removed prior to analysis. 3–4 whole coronal brain sections were analyzed per animal and respective brain region.

### Two-photon Imaging of Acute Brain Slices

300 µm thick acute coronal slices were prepared from 10 month old mice (3 mice per genotype) and stained with 0.001% Thiazine Red in aCSF for 10 min before imaging experiment. Imaging was performed with a two-photon laser scanning microscope directly coupled to a Chameleon ultrafast laser (Coherent). In order to create a discrete laser lesion, the laser was focused at −35 µm depths from the surface of the z-stack at a wavelength of 800 nm until autofluorescence was visible. In *APPPS1*-*Cx3cr1* ^+/gfp^ mice laser lesions were placed next to an Aβ plaque with a maximum distance to a neighboring Thiazine Red-positive plaque of 80 µm. For monitoring microglial responses 60 µm z-stacks were imaged with a step size of 3 µm covering a field of 307.2×307.2 µm every minute for 60 min. Recordings were analyzed with Image J MacBiophotonics, as adapted from Davalos *et al.* 2005 [2]. For calculating the average microglial response 7 (*Cx3cr1* ^+/gfp^) or 8 (*APPPS1*-*Cx3cr1* ^+/gfp^) ROIs from 4 mice per genotype were analyzed.

### In vivo Imaging

For intravital imaging, β-amyloid plaques were labeled with methoxy-XO4 (10 mg/kg) as described [27]. Methoxy-XO4 was kindly provided by William Klunk, University of Pittsburgh, USA. Before surgery mice were anesthetized by i.p. injection of a xylazine and ketamine mix in physiological saline (9.2 and 131 mg/kg body weight correspondingly). At approximately the center of the parietal bone a small cranial window with a 1.5–2 mm diameter (corresponding to an area of 1.8–3 mm^2^) was prepared using a high-speed dental drill. A circular bone fragment was carefully removed and the dura mater was left intact. Focal lesion and time-lapse recording was started 30 min after the surgical procedure to ensure absence of bleeding. All *in vivo* recordings were made in a time interval between 30 min and 8 h after the surgical procedure. The skull was fixed to the microscope stage and aCSF was placed into the chamber to allow imaging using a water-immersion objective (20x, NA 0.95, WD 2 mm; Olympus, Germany). Imaging was performed by a commercially available multiphoton imaging system (TriM Scope I, LaVision BioTec, Germany), equipped with a Ti:Sa laser (Chameleon Ultra II, Coherent). Fluorescence (GFP excitation wavelength 920 nm, methoxy-XO4 excitation wavelength 800 nm) was collected by three non-descanned PMT-detectors using dichroic mirrors and three interference filters (593/40 nm, 525/50 nm and 447/40 nm). XYZ-Stacks were collected every minute with a z-plane distance of 2 µm at a frequency of 400 Hz covering a depth range of 20 µm to 80 µm. The focal laser lesion was applied at a depth of 60 µm by steering the laser spot to the center of the field of view and irradiating for 300 ms with 50 mW energy at 920 nm. Microglial response was analyzed as described above with 8 (*Cx3cr1* ^+/gfp^) and 6 (*APPPS1*-*Cx3cr1* ^+/gfp^) ROIs from 3 mice per genotype.

### Statistical Analysis

Data sets were tested for normality by Shapiro-Wilks Test using SPSS. Two-sided levels of significance were determined by using the non-parametric Mann-Whitney-U- Test or the parametric T-Test according to the distribution and are depicted as *p<0.05, **p<0.01, ***p<0.001. Data are presented as mean ± s.e.m. For statistical analyses matching regions of interest (ROI) of multiple acute brain slices or cell cultures derived from various mice per experimental group were assessed as indicated and analyzed in a standardized fashion.

## Results

### Directed Microglial Process Motility Towards Acute Tissue Lesion is Impaired in Transgenic AD Mice

**Figure 1:**
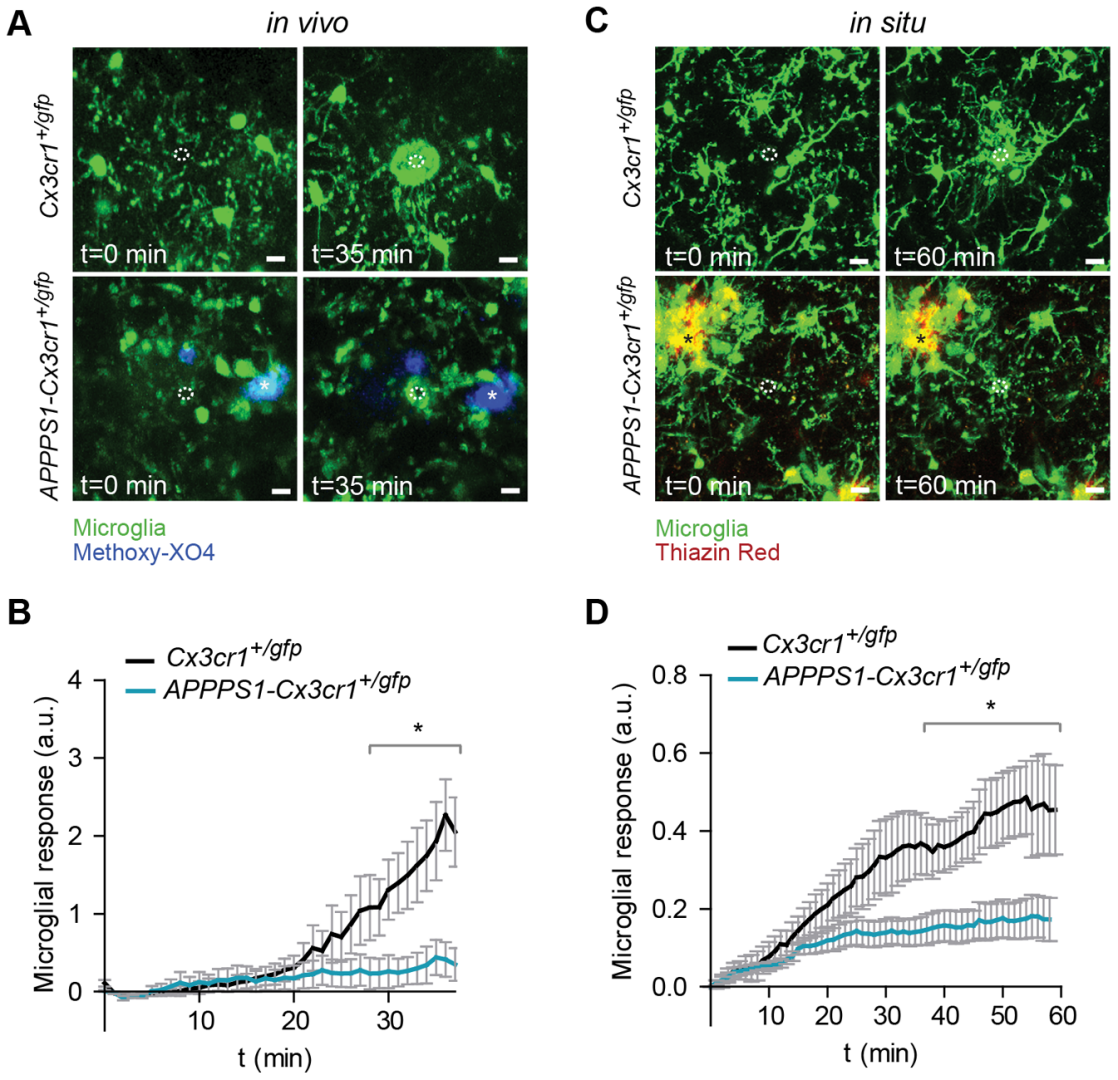
Lesion-directed microglial process movement is impaired in a mouse model of cerebral amyloidosis. (**A**) Representative intravital two-photon microscopy images and (**B**) time course of microglial process movement towards a laser-induced micro-lesion (dashed circle) in 8 month old live anaesthetized *APPPS1*-*Cx3cr1* ^+/gfp^ (n = 6) and *Cx3cr1* ^+/gfp^ mice (n = 8). Aβ plaques are stained with Methoxy-XO4 (blue, *). (**C**) Representative images and (**D**) relative microglial response to laser lesions in acute cortical cerebral slices of 10 month old *APPPS1*-*Cx3cr1* ^+/gfp^ (n = 8) and *Cx3cr1* ^+/gfp^ (n = 7) mice. Aβ plaques are stained with Thiazine Red (red, *). Data are mean ± s.e.m, *p<0.05. Scale bars: 10 µm. a.u. = arbitrary units.

Microglia respond to defined tissue injuries by extending their processes towards the lesion in an ATP-dependent fashion [2]. We used this canonical function of microglia as a measure to evaluate their functionality in an AD mouse model *in vivo* by intravital time-lapse two-photon microscopy. Notably, the ability of microglia for directed extension of their processes towards a lesion was largely reduced in Aβ plaque carrying *APPPS1-Cx3cr1* ^+/gfp^ mice (**Fig. 1A and B** **and Videos S1 and S2**), which were obtained by crossbreeding *APPPS1* mice, a mouse model of cerebral amyloidosis [21] with *Cx3cr1* ^gfp/gfp^ mice [23], a mouse model that allows visualization of microglia. Aβ plaques were labeled by intraperitoneal injection of Methoxy-XO4 [27]. Whereas microglia from 8 month old *Cx3cr1* ^+/gfp^ control animals moved their processes towards the laser-induced injury with an average peak response of 2.44±0.6 arbitrary units (a.u.), we only detected a sparse response of microglia in 8 month old *APPPS1-Cx3cr1* ^+/gfp^ mice, which harbor a substantial Aβ burden at that age (average peak response of 0.44±0.22 a.u., **Fig. 1A and B**). Since *in situ* experiments using acute cerebral slices allow stringent quantification of microglial response in a high number of experiments, we additionally conducted two-photon microscopy studies in acute coronal cerebral slices. Here, microglia exhibited slightly slower but otherwise similar process motility towards the lesion as compared to *in vivo* experiments. Importantly, microglial response towards the laser lesion in acute cerebral slices from 10 month old Aβ plaque-carrying *APPPS1-Cx3cr1* ^+/gfp^ mice were significantly reduced compared to their *Cx3cr1* ^+/gfp^ littermates **(** **Fig. 1C and D** **, Videos S3 and S4)**. Together, these data indicate that lesion-directed process extension as a typical functional feature of microglia is severely impaired in *APPPS1* mice that harbor Aβ plaques.

### Phagocytic Activity of Cortical Microglia is Impaired in APPPS1 and APP23 Mice

**Figure 2:**
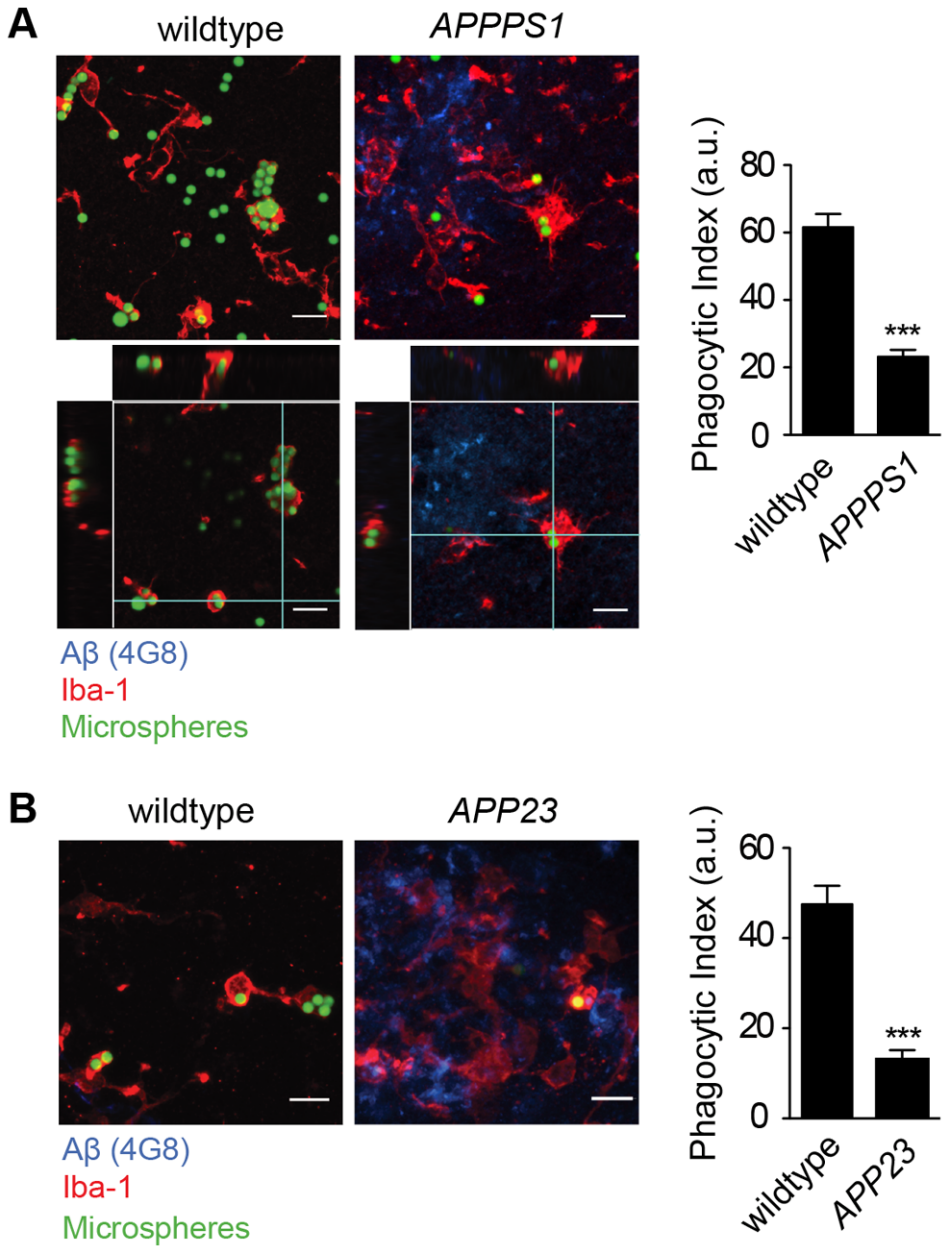
Phagocytic capacity of cortical microglia is impaired in two mouse models of cerebral amyloidosis. (**A**) Representative images (left) and microglial phagocytic index (in arbitrary units, a.u., right) of 9 month old *APPPS1* mice and wildtype littermate controls (3 mice per genotype; p<0.001). Images show microglia (Iba-1, red), Aβ (4G8, blue) and fluorescent microspheres (green). Orthogonal views of z-stack images are shown in the bottom panel. (**B**) Representative images (left) and microglial phagocytic index of 20 month old *APP23* and age-matched control mice (3 mice per genotype, p<0.001, right) are shown. Data are mean ± s.e.m, ***p<0.001. Scale bars: 10 µm.

We next aimed to investigate another key function of microglia - their phagocytic capacity. Since directed microglial process motility was similarly impaired when assessed intravitally or in acute cerebral slices derived from *APPPS1* mice, we reasoned that defined microglial functions are adequately reflected in acute cerebral slice preparations. As microglia in different brain regions, including cerebellum and hippocampus, can be readily evaluated with this *in situ* method, we used cerebral slice preparations to investigate microglial phagocytic capacity and quantified uptake of fluorescent polystyrene microparticles by microglia of *APPPS1* mice as described previously [25]. Likewise to the impaired directed process extension, microglial phagocytic activity in 9 month old *APPPS1* mice was significantly reduced when compared to wildtype littermates (**Fig. 2A**).

Mutations of PS1 have been suggested to influence the phagocytic activity of cells [28], [29]. To prove that functional decline of microglia is a general feature of AD pathology, and to exclude an impact of the PS1 transgene on microglia in *APPPS1* mice unrelated to amyloid pathology, we assessed microglial phagocytic activity in *APP23* mice, a transgenic mouse model of cerebral amyloidosis that is based on transgenic expression of APP with the Swedish mutation [22], but not of mutated PS1. Again, using *in situ* quantification of microglial phagocytosis, we detected a drastic impairment of microglial phagocytic activity in *APP23* mice (**Fig. 2B**). While we formally cannot exclude an – overall rather unlikely - impact of the APP-transgene on the performance of microglia from *APP23* mice, these data indicate that microglial dysfunction occurs in cerebral amyloidosis mouse models irrespective of the transgenic strain used and, thus, is dependent on cerebral amyloidosis.

### Impairment of Microglial Phagocytic Capacity Correlates Temporally and Spatially with the Appearance of Plaques

**Figure 3:**
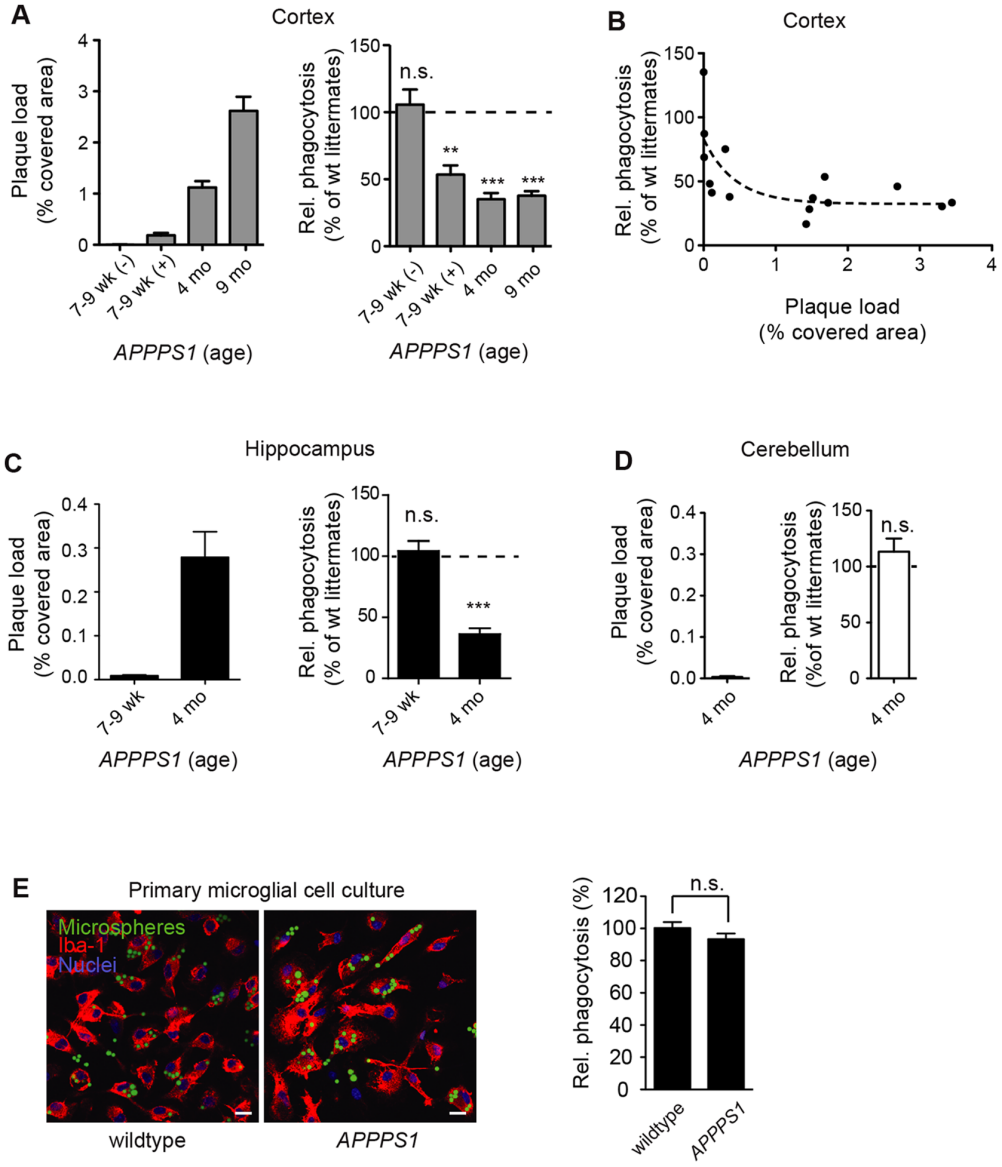
Impairment of microglial phagocytosis in *APPPS1* mice correlates with Aβ plaque deposition. (**A**) Aβ plaque load (brain area covered by Thiazine red-positive plaques) and relative microglial phagocytic activity normalized to corresponding wildtype littermate in the cortex of 7–9 week, 4 and 9 month old *APPPS1* mice. 7–9 week old mice were sub-classified according to apparent 4G8 positive plaque deposition as with (+) or without (−) detectable plaque load. (**B**) Correlation between extent of plaque load and relative microglial phagocytic activity in the cortex of *APPPS1* mice. (**C, D**) Thiazine red-covered area and relative phagocytic activity of microglia in the hippocampus of 7–9 week and 4 month old mice (**C**) and in the cerebellum of 4 month old *APPPS1* mice (**D**). Absolute values of microglial phagocytic indices from *APPPS1* mice were normalized to wildtype littermate controls (3–4 mice per age group and genotype, ***p<0.001). (**E**) Phagocytic index (3 independent experiments, p = 0.181) and representative images of primary microglial cultures from wildtype and *APPPS1* mice. Microglia (Iba-1, red), nuclei (DRAQ5, blue) and microspheres (green). All data are mean ± s.e.m, *p<0.05, **p<0.01. a.u. = arbitrary units. Scale bars: 10 µm.

To test whether the observed impairment of microglial phagocytosis depends on Aβ plaque deposition, we compared microglial phagocytosis in 7–9 week old *APPPS1* mice that had not yet developed detectable amyloid plaque burden to 7–9 week old *APPPS1* mice that showed first cortical amyloid plaques (**Fig. 3A** and **Fig. S1**), as detected by 4G8 immunohistochemistry or Thiazine Red staining. Importantly, in slices prepared from 7–9 week old *APPPS1* mice lacking cortical plaque load, microglial phagocytosis was as efficient as in wildtype littermates. However, we observed a significant reduction in phagocytosis in acute slices from 7–9 week old *APPPS1* mice that showed first cortical amyloid plaques (**Fig. 3A**). In slices from 4 month old *APPPS1* mice with significant cortical plaque load (**Fig. 3A** and **Fig. S1)**, impairment of microglial phagocytosis was already similar to 9 month old *APPPS1* mice (**Fig. 3A**). Overall, relative microglial phagocytic activity and cortical Aβ plaque burden exhibited a significant inverse correlation with a Spearman’s correlation coefficient of ρ (rho) = −0.75 (p = 0.0014, **Fig. 3B**).

To further establish the interconnection of Aβ plaque appearance and microglial dysfunction, we investigated microglial phagocytosis in various brain regions of *APPPS1* mice differing in the onset of plaque deposition [21]. In plaque-bearing cortices of 7–9 week old *APPPS1* mice, phagocytic activity was impaired, whereas it was normal in still plaque-free hippocampi of the same *APPPS1* animals (**Fig. 3C** and **Fig. S1**). Moreover, phagocytic capacity was impaired in hippocampi from 4 month old *APPPS1* mice, when plaques were present in that region. Importantly, in the cerebellum, which is devoid of plaques even in aged *APPPS1* mice ([21] and **Fig. S1**), phagocytic capacity remained unaltered also at 4 months of age (**Fig. 3D**).

Furthermore, we did not observe a significant difference in the phagocytic capacity of primary microglia derived from non-transgenic or *APPPS1* mice at postnatal day 1–3, i.e. prior to plaque deposition (**Fig. 3E**), indicating that impairment of phagocytosis is acquired in the presence of Aβ plaques over time.

### Aβ-plaque Load Reduction by Anti-Aβ Antibody Administration Restores Microglial Phagocytic Capacity in APPPS1 Mice

Finally, we investigated whether the functional impairment of microglia in plaque-bearing AD mice is reversible by interventions aimed at lowering Aβ burden. One way to counteract progressive Aβ plaque deposition is Aβ vaccination resulting in reduced plaque burden and increased number of Aβ-laden microglia in the cortex of AD patients [30]. Additionally, clearance of amyloid deposits and increase in microglial response was observed by *in vivo* imaging after stimulation with Aβ-specific antibodies when administered directly to an open skull preparation in a mouse model of AD [31].

**Figure 4:**
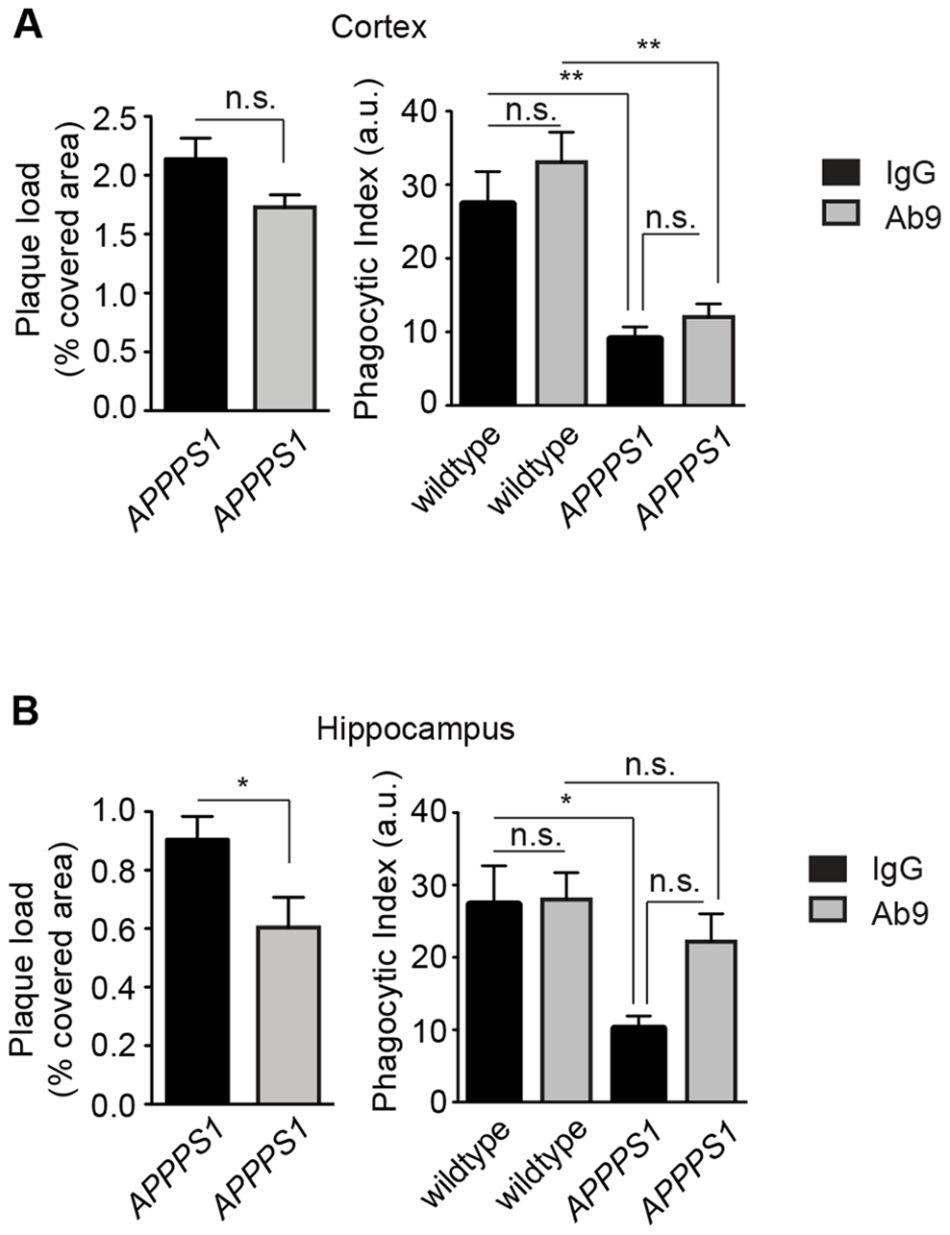
Passive anti-Aβ vaccination reduces plaque burden and restores hippocampal microglial phagocytic activity. 5 month old *APPPS1* mice (n = 3 mice per group) and wildtype littermates (n = 2 mice per group) were biweekly injected intraperitoneally with IgG (black bar) or anti-Aβ antibody (Ab9, grey bar) for 6 weeks. The area covered by Thiazine Red-positive Aβ plaques in cortex (**A**) and hippocampus (**B**) of 6.5 month old *APPPS1* mice or age-matched controls treated with IgG or Ab9 is shown in the left panel. Absolute values of microglial phagocytic indices in the cortex (**A**) and hippocampus (**B**) of the same mice are depicted on the right panel. All data are mean ± s.e.m, *p<0.05, **p<0.01. a.u. = arbitrary units.

Amyloid plaque burden was therefore reduced in 5 month old plaque-carrying *APPPS1* mice by passive Aβ vaccination administering the anti-Aβ antibody Ab9 [32] biweekly for six weeks. This treatment significantly diminished Thiazine Red-stained dense core plaques in the hippocampus of *APPPS1* mice, while there was no significant reduction of amyloid in the cortex when compared to control IgG-treated *APPPS1* littermates (**Fig. 4B** **left**, **4A left**). Strikingly, in accordance with the regional reduction in cerebral amyloid plaques, microglial phagocytic activity, measured in acute cerebral slices, was restored to levels found in Ab9- or IgG-treated wildtype mice exclusively in the hippocampus (**Fig. 4B** **right**). In contrast, microglial cells in the cortex, which lacked a substantial decrease in amyloid plaque burden, were still dysfunctional (**Fig. 4A** **right**). Thus, microglial dysfunction is not irreversibly impaired by Aβ plaque deposition, but can be restored during the course of disease.

## Discussion

The Aβ plaque-associated functional decline of microglia we have shown here may explain why temporary ablation of microglia for up to 30 days in *APPPS1* mice does not change the formation and maintenance of Aβ burden [19]. Our findings may also give an explanation as to why microglia fail to sufficiently remove Aβ plaques in AD *in vivo* [18], [20], and would support the notion of a dystrophic rather than an activated phenotype of microglia at late stages of AD [33].

Our methodological approach to quantify phagocytosis extends existing data on the function of AD-associated microglia during disease progression. It allowed us to overcome restrictions of current approaches including (i) the *ex vivo* analysis of isolated microglia whose functionality could be disturbed by the isolation procedure *per se*, (ii) post-mortem studies, which are stationary, typically restricted to defined time points, and do not provide direct information about cellular functionality or (iii) *in vivo* imaging studies that are typically limited to superficial cortical brain areas.

Mechanistically, Aβ may directly affect microglial function, as we were able to detect a significant inverse correlation between Aβ plaque burden and microglial phagocytic activity. Further studies will be required to address the question, of what kind of Aβ species may be responsible for inducing microglial dysfunction.

A large number of *in vitro* studies have shown that microglial cells release a battery of proinflammatory mediators, including nitric oxide and tumor necrosis factor alpha (TNFα), when stimulated with Aβ peptides [34]–[36]. Such chronic production of inflammatory molecules by microglia and/or constant exposure to a proinflammatory microenvironment within plaque-bearing AD brains may present another explanation of why microglial function may be influenced negatively [13], [37], [38]. This hypothesis is also supported by the finding that microglia stimulation with the proinflammatory cytokine TNFα leads to a downregulation of receptors involved in Aβ binding and degradation and reduces phagocytosis of Aβ *in vitro* [20].

To investigate a second biologically relevant microglial function we studied process motility by assessing process extensions towards an acute injury induced by a laser lesion [1], [2]. Our results obtained from *in vivo* observations were similar to data retrieved by us in acute brain slices with respect to speed and quantity of lesion-directed process movements. The significant decrease in the amount of microglial processes sent towards the injury site, which we consistently found both *in vivo* and *in situ*, supports a report on impaired baseline dynamics of plaque-associated microglia [27]. However, another detailed analysis of baseline microglial process turnover in *APPPS1* mice has shown that turnover speed of processes in microglia near plaques (within 50 µm from the plaque surface) was similar compared to microglia in non-transgenic animals. Only process speed in microglia directly on Aβ plaques was slightly but significantly reduced [9].

Notably, we show that functional impairment of microglial phagocytosis can be reversed to non-diseased aptitude by reducing amyloid by Aβ vaccination. This speaks against microglial senescence as the main underlying mechanism for the decline in microglial function demonstrated herein. Microglial senescence has been described in the human brain during aging and in Alzheimer’s disease and is associated with telomere shortening and reduced telomerase activity [39]. In contrast to the microglial phenotype reported here, cellular senescence is irreversible, at least in lymphocytes and at late stages of cellular senescence [40]. However, similar mechanisms might be involved in inducing microglial dysfunction and have to be investigated in future studies. Furthermore, microglial senescence may play an important role in humans during aging.

These data highlight the interrelation of plaque deposition and microglial behavior, thus expanding the possible modes of action of other amyloid-reducing approaches to rescuing microglial function. Such view is in line with a recent report on bexarotene-mediated reduction of Aβ burden, which resulted in an improvement in neuronal function and, importantly, increased the number of Aβ-laden microglia [41].

Taken together, our data demonstrate that microglial dysfunction develops early in the course of AD in an Aβ-dependent fashion and can be restored by interventional anti-Aβ approaches, such as Aβ vaccination. Our findings therefore suggest that novel treatment strategies aimed at maintaining or increasing microglial function may represent an attractive therapeutic approach even at advanced stages of AD.

## Supporting Information

Figure S1 Age- and brain area-dependent Aβ plaque load in *APPPS1* mice. Representative confocal images of fixed and stained acute coronal brain slices from *APPPS1* mice of the indicated age showing cortex (**a**), hippocampus (**b**) or cerebellum (**c**). Microglia (Iba-1, red), Aβ plaques (Thiazine Red, green) and nuclei (Hoechst 33258; blue). Scale bars: 50 µm.
(TIF)

Video S1 Rapid microglial response toward an acute laser lesion in *Cx3cr1* ^+/gfp^ mice. Intravital time-lapse two-photon microscopy over a period of 36 min shows rapid microglial response upon a laser lesion in 8 month old *Cx3cr1* ^+/gfp^ mice.
(AVI)

Video S2 Impaired microglial response toward an acute laser lesion in *APPPS1*-*Cx3cr1* ^+/gfp^ mice. Intravital time-lapse two-photon microscopy over a period of 54 min shows attenuated response of microglial processes (green) toward a laser lesion in 8 month old *APPPS1*-*Cx3cr1* ^+/GFP^ mice. Aβ plaques are stained with Methoxy-XO4 (blue).
(AVI)

Video S3 Rapid microglial response to a laser-induced lesion in an acute cerebral slice from *Cx3cr1* ^+/gfp^ mice. Time-lapse two-photon microscopy of an acute cerebral slice from 10 month old *Cx3cr1* ^+/gfp^ mice over a time period of 60 min displays rapid microglial process accumulation around a laser-induced lesion.
(AVI)

Video S4 Microglial response to a laser-induced lesion is impaired in acute brain slices from *APPPS1*-*Cx3cr1* ^+/gfp^ mice. Time-lapse two-photon microscopy of an acute cerebral slice from 10 month old *APPPS1*-*Cx3cr1* ^+/gfp^ mice over a period of 60 min shows impaired accumulation of microglial processes around a laser-induced tissue lesion. Aβ plaques are stained with Thiazine Red (red).
(AVI)
