# Supplementary figures and images for: Functional Impairment of Microglia Coincides with Beta-Amyloid Deposition in Mice with Alzheimer-Like Pathology
Source: PLoS One. 2013 Apr 8;8(4):e60921. doi: 10.1371/journal.pone.0060921 (PMC3620049; doi:10.1371/journal.pone.0060921)

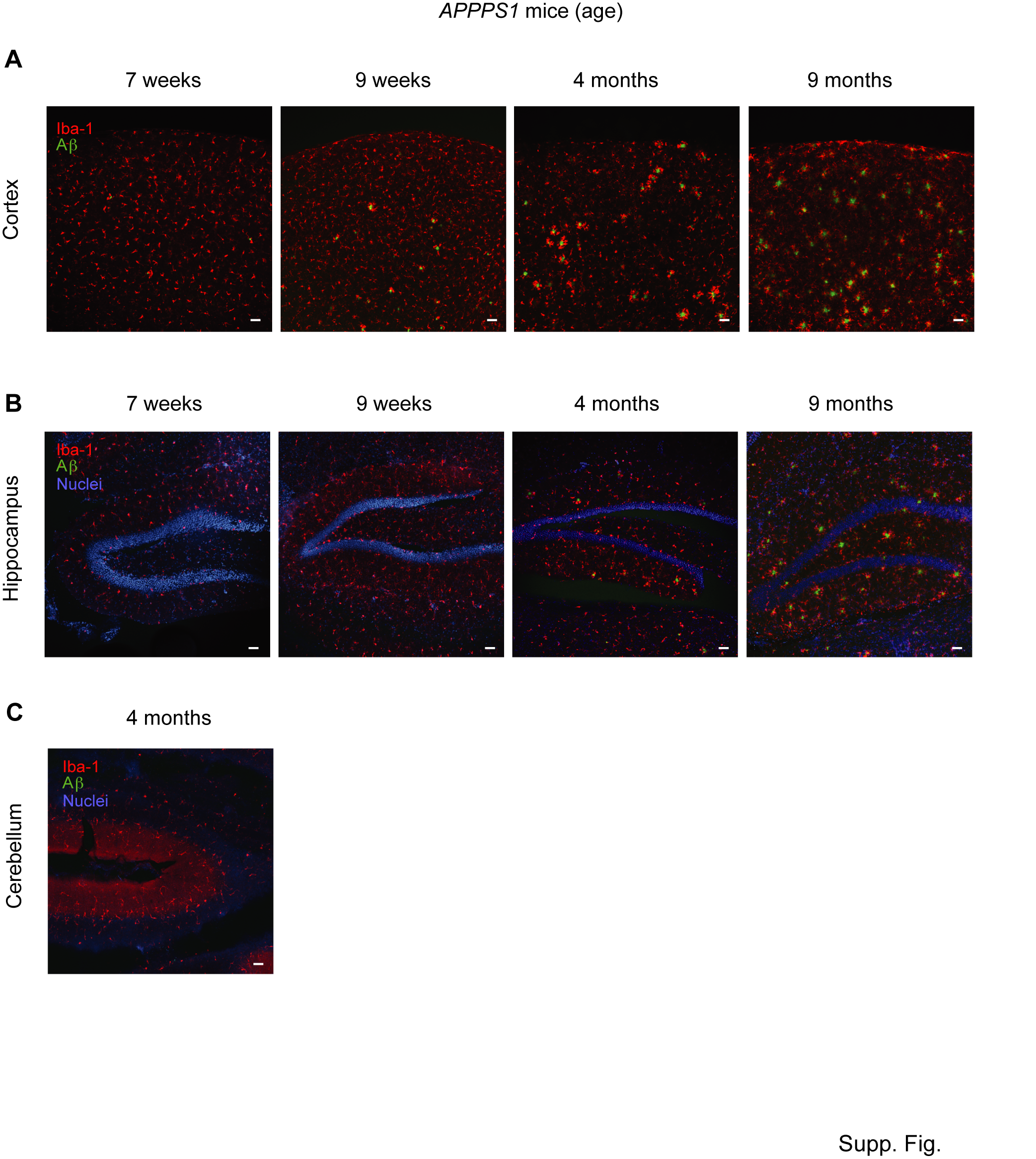

Supplement: Figure S1 — Age- and brain area-dependent Aβ plaque load in APPPS1 mice. Representative confocal images of fixed and stained acute coronal brain slices from APPPS1 mice of the indicated age showing cortex (a), hippocampus (b) or cerebellum (c). Microglia (Iba-1, red), Aβ plaques (Thiazine Red, green) and nuclei (Hoechst 33258; blue). Scale bars: 50 µm. (TIF) [file pone.0060921.s001.tif]
